# Chronic, acute and protocol-dependent effects of exercise on psycho-physiological health during long-term isolation and confinement

**DOI:** 10.1186/s12868-022-00723-x

**Published:** 2022-06-30

**Authors:** V. Abeln, E. Fomina, J. Popova, L. Braunsmann, J. Koschate, F. Möller, S. O. Fedyay, G. Y. Vassilieva, S. Schneider, H. K. Strüder, T. Klein

**Affiliations:** 1 Institute of Movement and Neurosciences, Center for Health and Integrative Physiology in Space (CHIPS), German Sport University Cologne, Am Sportpark Muengersdorf 6, 50933 Cologne, Germany; 2 Institute of Biomedical Problems (IBMP), Russian Academy of Sciences, Khoroshevskoye shosse 76A, 123007 Moscow, Russia; 3 Geriatric Medicine, School of Medicine and Health Sciences, Carl Von Ossietzky University Oldenburg, Ammerlaender Heerstr. 140, 26129 Oldenburg, Germany; 4 Department of Exercise Physiology, German Sport University Cologne, Am Sportpark Muengersdorf 6, 50933 CologneCologne, Germany; 5 Institute of Sport Science, University of Rostock, 18057 Rostock, Germany

**Keywords:** Mental Health, Cortisol, Cognitive performance, Mood, Affect, Physical Activity, Neurotrophic Factors

## Abstract

Exercise could prevent physical and psychological deteriorations, especially during pandemic times of lock-down scenarios and social isolation. But to meet both, the common exercise protocols require optimization based on holistic investigations and with respect to underlying processes. This study aimed to explore individual chronic and acute effects of continuous and interval running exercise on physical and cognitive performance, mood, and affect and underlying neurophysiological factors during a terrestrial simulated space mission. Six volunteers (three females) were isolated for 120 days. Accompanying exercise training consisted of a continuous and interval running protocol in a cross-over design. Incremental stage tests on a treadmill were done frequently to test physical performance. Actigraphy was used to monitor physical activity level. Cognitive performance, mood (MoodMeter^®^), affect (PANAS), brain-derived neurotrophic factor (BDNF), insulin-like growth factor 1 (IGF-1), vascular-endothelial growth factor (VEGF), and saliva cortisol were investigated prior to, four times during, and after isolation, pre- and post-exercise on two separate days, respectively. As a chronic effect, physical performance increased (and IGF-1 tended) in the course of isolation and training until the end of isolation. Subjective mood and affect state, as well as cognitive performance, basal BDNF and VEGF levels, were well-preserved across the intervention. No acute effects of exercise were detected, besides slower reaction time after exercise in two out of nine cognitive tests, testing sensorimotor speed and memory of complex figures. Consistently higher basal IGF-1 concentrations and faster reaction time in the psychomotor vigilance test were found for the continuous compared to the interval running protocol. The results suggest that 120 days of isolation and confinement can be undergone without cognitive and mental deteriorations. Regular, individual aerobic running training supporting physical fitness is hypothesized to play an important role in this regard. Continuous running exercise seems to trigger higher IGF-1 levels and vigilance compared to interval running. Systematic and prolonged investigations and larger sample size are required to follow up on exercise-protocol specific differences in order to optimize the exercise intervention for long-term psycho-physiological health and well-being.

**Supplementary Information:**

The online version contains supplementary material available at 10.1186/s12868-022-00723-x.

## Introduction

Living under isolated, confined, and extreme (ICE) conditions comes with a number of potential factors endangering physical as well as psychological performance and health. While living in isolation has gained high attention during the recent COVID-19 pandemic, it has concerned space research already for a long time. High-level cognitive performance is fundamental for astronauts during space missions and may have severe consequences when impaired attention, working memory or problem-solving ability occurs. A positive and stable psychological condition in terms of mood and affect is at least equally important when crewmembers need to live, work and function together for a long time under extreme conditions [1]. But factors, timing and potential interventions for mental health are equally important for people on Earth considering the rising numbers of mental health impairments.

Affect describes feeling states like emotions and moods and plays an important role in regulating cognition, behavior, and social interactions [2]. Positive emotions, self-perception and -regulation beneficially contribute and support coping of stressful experiences [3], and thus is suggested to support living in ICE. The link between mood, affect and cognitive performance highlights the need to respect and conserve all these to an equal extent during long-term missions. Space flight and long-term isolation missions repeatedly detected degradations in problem-solving [4], working memory [5], attention [6] as well as mood and affect [1] even though crewmembers have been psychologically screened during recruitment. But the reports are contradictory, and data considering changes in cognitive performance in conjunction with changes in mood and affect is still insufficient in order to uncover influencing factors for individual psychological outcomes and efficient interventions.

Exercise is a promising intervention for physical degradation but also both psychological domains (cognition and mood/affect) [7, 8]. Being aware of the holistic exercise benefit, space and space analogue missions are mostly designed with exercise as a mandatory intervention. Missions abstaining from exercise interventions are rare, and inactive control groups are widely missing. But some references emphasize the significance of regular physical exercise by differentiating between active or sedentary crewmembers during Antarctic overwintering [9] and ambulatory control groups that stopped their habitual exercise routine [10, 11]. This might explain why some previous space-related studies with exercise intervention did not find impairments. But it also leaves the assumption that exercise prescriptions are not (always) optimized and possibly not individualized enough so far, as impairments are still commonly reported.

Literature regarding the most beneficial exercise intervention for mood and affect state is contradictory. A meta-analysis conducted by Niven et al. [12] comparing both interval and continuous exercise of high and moderate intensity concludes that the majority of studies report better affect after moderate continuous exercise (mean difference − 0.72, 95% CI − 1.64 to 0.20, p = 0.12) and five to twenty minutes after exercise (MD − 0.22, 95% CI − 0.40 to − 0.04, p = 0.02). However, the authors report about a large degree of heterogeneity in the reviewed studies. Similarly, studies comparing continuous to interval exercise of different intensities in regard to their effect on cognitive performance are also mixed, but the majority demonstrates a difference between these exercise modes in facilitating inhibitory control [13–15]. In addition higher arousal was found following high-intensity interval compared to moderate continuous exercise [12]. Interval exercise has been discussed to accelerate oxygen uptake kinetics slightly more than continuous endurance training [16, 17], which chronically might support cognitive performance. However, it remains unclear whether the effect of exercise on mood, affect, and cognition is dependent on the intensity or on the modality (interval vs. continuous). Accordingly, more studies are required which aim to test different exercise protocols such as intensity-matched continuous versus interval running exercise.

Considering potential underlying factors and mechanisms, exercise was shown to trigger hormonal responses supporting brain plasticity and eventually brain function, cognition, and psychological health. In animal studies, the brain-derived neurotrophic factor (BDNF), insulin-like growth factor 1 (IGF-1), and vascular-endothelial growth factor (VEGF) have been identified as important markers of exercise-induced support for brain function and health, and the bridge between peripheral and central changes and towards human models has been built [18, 19]. Reductions of BDNF messenger ribonucleic acid in the brain with impaired memory consolidation were found during social isolation [20, 21], and BNDF was associated with psychological and psychiatric disorders, like depression [22]. Exercise is known to trigger BDNF release (for review: [23–26]). IGF-1 mediates neurogenesis and modulates brain activity, including cognitive functions [27]. Exercise was also found to elevate serum IGF-1 levels [27], and there is a large body of evidence for its correlation with fitness (for review: [28]). VEGF has been shown to play a role in exercise-induced adult hippocampal neurogenesis and cognitive improvements (for review: [29]). Despite controversial findings in the literature, human studies and reviews provide consistent evidence that aerobic exercise enhances these neurotrophic and growth factors [29–34]. In previous investigations, decreasing BDNF and increasing IGF-1 level was found during isolation with regular exercise, whereas non-isolated and non-exercising control participants during 60 days of bedrest with or without artificial gravity exposure did not show any changes [10, 11, 35]. Therefore, neurotrophic factors are suggested play a role for isolation- and exercise-dependent changes.

Another potential underlying factor with consequences on brain health, function, and morphology is a disrupted hormonal stress regulation, indicated by changed cortisol levels or profiles, which have been detected in ICE missions [36, 37]. Cortisol can cross the blood–brain barrier and bind to receptors within the hippocampus, amygdala, and frontal lobe and high levels of free cortisol was found to negatively influence cognitive performance, e. g. learning and memory [38, 39]. Stress was shown to alter brain structure and function and hippocampal release of BDNF as well as VEGF [40, 41], and regular exercise is suggested to positively influence cortisol regulation [42, 43]. Some shorter duration isolation studies with accompanying exercise could show elevated cortisol concentrations without related cognitive or mood impairments [10, 11, 44]. But higher chronic cortisol levels were also found for high-density (five days per week) compared to less dense exercise protocols in isolation (three to four times per week) compared to non-isolated and non-exercising control groups [10, 11], which warrants further investigation.

Consequently, exercise, in particular an aerobic exercise protocol, seems to be a potent comprehensive intervention to meet the challenging effects of ICE environments on psycho-physiological performance and health, but different exercise protocols need to be investigated for optimization and individualization. This project aimed to clarify the chronic and acute effects of intensity-matched continuous and interval running exercise on physical and cognitive performance, mood, and affect. It intends to explore underlying neurophysiological factors and to provide additional (widely-missing) gender-specific data during long-duration missions under ICE conditions.

## Materials and methods

### Design

The study was implemented in the Scientific International Research in Unique Terrestrial Station 2019 (SIRIUS-19) isolation mission. The mission was conducted from March to July 2019 in the Nezemnyy Eksperimental'nyy Kompleks (NEK) multicompartment facility of the Institute of Biomedical Problems, Russian Academy of Sciences (IBMP RAS) in Moscow under joint operation with the National Aeronautics and Space Administration Human Research Program (NASA HRP). Detailed information about the SIRIUS-19 mission scenario can be found at: https://www.nasa.gov/sites/default/files/atoms/files/sirius_19_booklet.pdf. The German Space Agency participated amongst other partner countries according to the general conception developed by IBMP and NASA HRP.

A six-headed crew of three Russian women (mean age 29.7 ± 2.1 years), one Russian and two American men (mean age 33.7 ± 6.4 years) were isolated for 122 days under space-analogue conditions simulating a mission to the Moon and extravehicular activities on a lunar surface simulator. Mission scenario was laid out to mimic a real space-mission as far as possible by embedding similar daily schedules, food, workload and tasks, timeline of communication, stressors, isolation and confinement conditions (closed module, limited access to media), and physical exercise training.

**Fig. 1:**
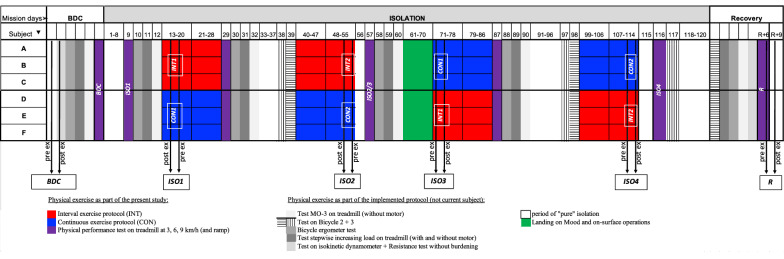
Schematic of test design and timeline of physical training. Exercise as subject of the current study is indicated in red (interval exercise, INT) and blue (continuous exercise, CON) or violet (physical performance tests). Other physical exercise tests are indicated in grey or stripped. Black arrows indicate the assessment days (BDC: baseline data collection, ISO1-4: four quarter during isolation, R: recovery period after isolation) either pre- or post-exercise. White areas indicate phases of “pure” isolation without physical tests or training. The green area is showing the period when landing on the Lunar surface and on-surface operations took place

The physical exercise training protocol was designed by IBMP RAS in a joint agreement with the German Sport University. A physical performance test was conducted prior to isolation with stepwise increasing load on a treadmill until exhaustion to determine the metabolic costs, maximal heart rate, maximal oxygen uptake and maximal velocity. Training during isolation prescribed running exercise on a treadmill following either an interval (INT) or a continuous (CON) training protocol in a cross-over design with a “wash-out period of fifteen days in-between (for details, see Fig. 1 and additional file 1). Running exercise was executed on three consecutive days followed by one day of rest. This four-day micro cycle was repeated four times. After a break of 12 to 15 days the same four times four-day cycle was repeated (total of 24 training days per protocol). CON was performed at a consistent speed at 50% of individual maximum heart rate, and INT protocol alternated between running and walking intervals between 30 to 80% of maximal heart rate (average speed day 1 of the micro cycle: 47% of individual maximum heart rate, day 2: 43%, day 3: 44%). Both protocols were executed on average at 7.5 km/h ± 0.55 km/h treadmill velocity and for a duration of 30 to 40 min (see appendices). Exercise intensity was regulated via treadmill speed and heart rate monitors (Polar M400, Polar Electro GmbH, Büttelborn, Germany).

### Data acquisition

Data of the present investigation was collected at the following timepoints: during baseline data collection (BDC) between days − 14 and − 7 prior to isolation, at the beginning and end of the INT or CON exercise protocol, on mission day (MD) 14 to 16 (hereafter referred to as ISO1), on MD53/54 (ISO2), MD79/80 (ISO3) and MD110/112 (ISO4) during isolation, as well as during the recovery phase (R) after isolation between day R + 6 to R + 9. The data collection was conducted before exercise or on a day without exercise (pre-exercise) and on a separate day immediately after exercise (post-exercise) at each timepoint in alternating order. All crewmembers were instructed, familiarized, and trained by the investigators prior to BDC. Assessments during BDC and R were conducted autonomously but under supervision. During isolation, crewmembers were carrying out the assessments autonomously in pairs.

### Physical activity data

#### Performance tests

Physical performance tests on treadmill were performed on BDC-15 (± 12), on MD9 (± 1) hereafter referred to as ISO1, MD57 (± 1, ISO2/3), MD117 (± 1, ISO4) as well as on R + 6. Additional tests have been performed on MD29 and 87, which are not subject of this manuscript. The test commenced with a moderate part running at 3 km/h for 5 min. It continued with a 10 min period of intervals changing between 3 and 6 km/h velocity and then stages of five minutes each at 3, 6 and 9 km/h. It ended with an incremental part (ramp), which started with 3 km/h for 60 s, where the speed was increased every 30 s by 1 km/h, until the participant was not able to follow the speed of the treadmill belt. Only the five min stages at 3, 6 and 9 km/h are subject of the present study.

### Heart rate, lactate, VO_2peak_, and RER_peak_

Heart rate and lactate concentration are two important parameters used during physical performance tests and are known to decrease with increasing levels of physical fitness at the same work rate. Heart rate was continuously recorded, but only the last 30 s were averaged to calculate a representative heart rate for each stage. 20 μl capillary blood from the fingertip was collected during the last 30 s of the 3, 6 and 9 km/h stages for blood lactate analysis (Biosen C_Line—EKF-diagnostic, Barleben, Germany). As part of another project, peak oxygen uptake (VO_2peak_) and peak respiratory exchange ratio (RER_peak_) resulting from these physical performance tests are included here to support the physical performance outcome. Parts of this data and additional variables have been published by Koschate et al. [17]. Respiratory data was collected using a spiro-ergometry system (Metalyzer 3B, Cortex Medical Systems, Leipzig, Germany). For VO_2peak_ and RER_peak_, data during the last 30 s before the termination of the physical performance test (end of ramp) were averaged.

### Actigraphy

ActiGraph GT3X + accelerometers (ActiGraph, Pensacola, Florida) were used to monitor daily physical activity. Participants wore the device on the non-dominant wrist continuously throughout the intervention. Three-dimensional accelerations were recorded in 10 s epochs. Raw data was exported using ActiLife (version 6; ActiGraph, Pensacola, Florida), and acceleration signals were post-processed using the normal filter of the ActiLife software. Step counts, based on accelerometer data collected on the vertical axis, were analyzed for each day. days were excluded from further analysis if more than one hour of data was missing per day. Two participants were excluded from the actigraphy analysis due to largely missing data in isolation.^1^ Valid data of mean steps per day from the remaining four subjects were averaged for BDC (13 ± 5 days), for the time in isolation (first half (ISO1-2): 19 ± 4 days; second half (ISO3-4): 41 ± 6 days, and for the recovery phase (R): 5 ± 3 days).

#### Growth factors (BDNF, IGF-1, VEGF)

The medical doctor of the crew carried out venipuncture on the basilic vein for pre-exercise assessments in the morning (mean time 07:58 ± 0:14 am) after at least eight hours of fasting and for post-exercise assessment about 5 min after termination of exercise (mean time 02:34 ± 2:42 pm) respectively. After 30 min in an upright position, samples were centrifuged at 2.500*g* for ten minutes. Serum aliquots were stored at − 80 °C until the end of the mission and shipped from Moscow to Germany under temperature-controlled conditions below − 20 °C. Serum levels of IGF-1, VEGF, and BDNF were measured using commercial enzyme-linked immunosorbent assays (ELISAs) kits (R&D Systems, Inc., Minneapolis) according to the manufacture's protocols (lower limit of quantification & intra-assay coefficient for BDNF: > 0.001 ng/ml & 2.87 ng/ml; IGF-1 = 0.010 ng/ml & 4.3 ng/ml; VEGF < 5.0 pg/ml & 5.43 pg/ml). Resulting BDNF and IGF-1 concentrations are indicated in ng/ml, and VEGF in pg/ml.

#### Saliva cortisol

Salivary cortisol was shown to reliably reflect (total) plasma and free circulating cortisol concentrations (Levine, Zagoory-Sharon, Feldman, Lewis, & Weller, 2007). On data acquisition timepoints, saliva sampling was performed eight times in two-hour intervals throughout the day starting from 8 am to 10 pm using Salivette^®^ (Sarstedt, Nümbrecht, Germany). Samples were stored at − 20 °C and shipped together with the serum samples to Germany. Samples were defrosted and centrifuged for two minutes at 1.000 g. Analyses of saliva cortisol concentrations were performed using the DRG Salivary Cortisol ELISA kit (DRG Instruments GmbH, Marburg, Germany) following the provided instruction of the manufacturer (lower limit of quantification & intra-assay coefficient: 0.09 ng/ml & 3.9 ng/ml). Resulting concentrations are provided in ng/ml. In addition to the mean cortisol concentrations averaged over all participants per sample time (daytime) per assessment day, the area under the curve of the day profiles has been calculated as sum of rectangles.

#### Mood

The MoodMeter^®^ [45] was developed to detect short-term changes of the perceived physical state (PEPS), psychological strain (PSYCHO), and motivational state (MOT). It includes a short version of the "Eigenzustandsskala" [46]. It was presented as a paper–pencil version in participants' native languages containing 32-adjectives in mixed order, which had to be completed on a six-point Likert scale (0 = not at all, 5 = totally, the higher the score, the better the perceived state) based on their current feeling. This questionnaire has been used in various space-analogue studies, and details about the scoring have previously been published [9].

#### Positive and negative affect state (PANAS)

The self-report Positive and Negative Affect Scale (PANAS) [47] was implemented as part of NASA HRP Standard Measures in native language [48]. Twenty adjectives in relation to positive and negative affect were listed in alternating order and had to be rated on a 5-point Likert scale (1 = very slightly/not at all, 5 = extremely) based on individuals feeling and emotions during the past week. The ten statements to positive items were summed to the General Positive Affect Sum; for negative items, likewise to the General Negative Affect Sum. Accordingly, the higher the total sum between 10 and 50 is, the stronger is the respective perceived state of affect. Both sums have been identified in both intra- and inter-individual analyses and emerge in a consistent way across sets, time frames, response formats, language, and culture. The PANAS was completed on BDC-2, MD12, 53, 82, 110 and R + 11.

#### Cognition test battery

Cognition data were collected and provided by the NASA Behavioral Health & Performance Laboratory, Johnson Space Center, as part of Generalizability of Operational Feasibility and Acceptability of HFBP Exploration Measures in NEK project, supported by NASA's HRP. A detailed description of the cognitive test battery has been published [49, 50]. The following tests were performed in the order listed below and included: The Motor Praxis Task (MPT) measuring sensorimotor speed, the Visual Object Learning Task (VOLT) measuring memory for complex figures, the n-Back (NBACK) test measuring the working memory system, the Abstract Matching (AM) test measuring abstraction and flexibility components of executive function, the Line Orientation Test (LOT) measuring spatial orientation, the Emotion Recognition Task (ERT) measuring facial emotion recognition, the Matrix Reasoning Test (MRT) measuring abstract reasoning, the Digit-Symbol Substitution Task (DSST) measuring complex scanning, visual tracking and working memory, and the Psychomotor Vigilance Test (PVT) measuring sustained attention and reaction time. Participants were familiarized with the tests during BDC and repeated the battery in total three times during BDC, twenty-nine times during isolation and six times during R. However, only the data matched to the assessment days of the present study are used in this manuscript. Similar to Basner et al. [49], reaction time (in ms) was used as outcome variable for MPT, VOLT, NBACK, LOT, ERT, DSST, and PVT. Accuracy was calculated by dividing the number of correct responses by the number of stimuli for each task respectively, but MPT and PVT. For PVT number of lapses was used instead of accuracy.

### Statistics

Due to the small number of participants, non-parametric tests have been performed for statistical analysis. Only two physical training sessions of one subject due to illness were missed. Friedman ANOVA was used to calculate the chronic effects of *TIME/isolation* between BDC, ISO1, ISO2, ISO3, ISO4 and R. For physical performance test parameters (VO_2peak_, RER_peak_, lactate, heart rate), *TIME* effects were calculated between BDC, ISO1, ISO2/3, ISO4 and R. For physical activity (mean steps per day) BDC was compared to the first (ISO1-2) and second half (ISO3-4) of isolation and R. For the calculation of *TIME* effects, only pre-exercise data were used in order to exclude acute exercise effects and for standardized blood draw times and conditions. For Bonferroni correction of multiple comparisons, alpha-value was divided by the number of comparisons (= 15) and significance threshold set at p = 0.003, for lactate and heart rate (= 10) p = 0.005. In case of a significant *TIME* effect, Wilcoxon Test for paired samples was used. Wilcoxon Test was also used to determine differences between dependent variables: Exercise training protocol INT and CON (*PROTOCOL* effect), for which the latest data conducted during the INT training period (INT2) has been compared to the latest data during the CON training period (CON2). In order to test for carry-over effects between training protocols, the differences between states at the start of training protocols (INT1 versus CON1) were calculated by Wilcoxon test. Acute effects of exercise comparing all pre-exercise data with all post-exercise data (*acuteEXERCISE* effect) were calculated by Wilcoxon Test. For cortisol only, last samples of the day profile collected before and the first after exercise were used for the *acuteEXERCISE* effect. For Wilcoxon Tests, the level of significance was p = 0.05. Due to the small sample size, a gender-specific analysis was not possible, but data is presented descriptively per individual and sex in order to provide additional and gender-related information.

## Results

### Actigraphy

**Fig. 2:**
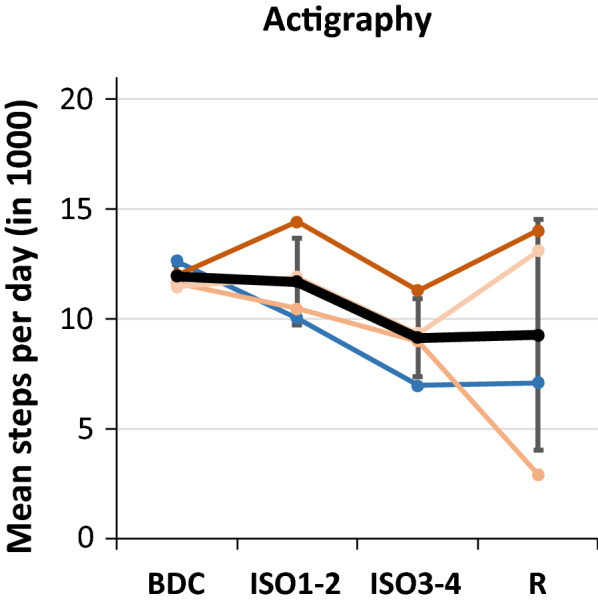
Results of actigraphy: Mean steps per day for four individuals (blue male, red females) and mean (black) for periods during baseline data collection (BDC), during the first two (ISO1-2) and last two (ISO3-4) quarters of isolation and during the recovery phase (R). Error bars indicate standard deviation

Mean steps per day tended to decrease in the course of the mission with high variance during R. The *TIME* effect did not reach the level of significance (see Table 1 , Fig. 2) but approaches it if R was excluded (p = 0.049). *PROTOCOL* effects were not calculated due to reduced data sets.

**Table 1:** Results of Friedman ANOVA testing the main effect of TIME, of Wilcoxon Test for main effects of PROTOCOL and acuteEXERCISE in columns, different variables are listed in rows

	Time		Protocol (INT vs. CON)				INT1 vs CON1	
	p	Chi^2^	p	Z			p	Z
Lactate (mmol/l) 0 km/h	0.001*	18.400	0.686	0.405			0.463	0.734
3 km/h	0.001*	18.400	0.917	0.105			0.028	2.201
6 km/h	0.006	14.267	0.225	1.214			0.917	0.105
9 km/h	0.008	13.733	0.345	0.943			0.028*	2.201
HEART RATE (beats/min) 0 km/h	0.000*	27.571	0.917	0.105			0.075	1.782
3 km/h	0.000*	26.143	0.345	0.943			0.075	1.782
6 km/h	0.002*	26.071	0.753	0.314			0.249	1.153
9 km/h	0.005*	18.786	0.075	1.782			0.028*	2.201
VO_2_ peak	0.106	7.630	1.000	0.000			0.046*	1.992
RER	0.003*	16.370	0.875	0.157			0.046*	1.992

	Time		Protocol (INT vs. CON)		Exercise (pre vs. post)		INT1 vs CON1	
	p	Chi^2^	p	Z	p	Z	p	Z
PANAS positive affect	0.978	0.781	0.465	0.730	0.153	1.429	0.144	1.461
PANAS negative affect	0.649	3.333	n/A	n7A	0.655	0.447	n/a	n/a
MOODMETER PEPS	0.822	2.192	0.594	0.533	0.973	0.034	0.249	1.153
PSYCHO	0.069	10.226	0.308	1.019	0.085	1.724	0.893	0.135
MOT	0.270	6.389	0.328	0.978	0.864	0.171	1.000	0.000
COGNITION MPT Reaction time	0.008	15.714	0.753	0.314	0.028*	2.201	0.893	﻿0,135
VOLT Reaction time	0.005	17.000	0.249	1.153	0.028*	2.201	0.893	0.135
VOLT Accuracy	0.776	2.500	0.173	1.363	0.529	0.629	0.201	1.278
NBACK Accuracy	0.531	4.129	0.590	0.539	0.753	0.314	0.500	0.674
NBACK Reaction time	0.093	9.428	0.753	0.314	0.753	0.314	0.500	0.674
AIM Accuracy	0.937	1.280	0.787	0.270	0.787	0.270	0.893	0.135
LOT Accuracy	0.705	2.971	0.686	0.405	0.600	0.524	0.917	0.105
LOT Reaction time	0.040	11.629	0.463	0.734	0.463	0.734	0.345	0.943
ERT Accuracy	0.055	10.823	0.834	0.210	0.173	1.363	0.917	0.105
ERT Reaction time	0.001*	21.229	0.753	0.314	0.753	0.314	0.753	0.314
MRT Accuracy	0.096	9.335	0.418	0.809	0.600	0.524	0.686	0.405
DSST Accuracy	0.815	2.238	0.068	1.826	0.173	1.363	1.000	0.000
DSST Reaction Time	0.001*	21.800	0.600	0.524	0.249	1.153	0.600	0.524
PVT Lapses	0.472	4.558	0.423	0.802	0.500	0.674	0.180	1.342
PVT Reaction time	0.709	2.943	0.028*	2.201	0.249	1.153	0.249	1.153
BDNF [ng/ml]	0.217	7.048	0.084	1.726	0.440	0.771	0.116	1.572
IGF-1 [ng/ml]	0.015	14.095	0.003*	2.981	0.052	1.943	0.753	0.314
VEGF [pg/ml]	0.115	8.857	0.347	0.941	0.076	1.771	0.600	0.524
CORTISOL (ng/ml) 08:00	0.167	7.810	0.583	0.549	0.116	1.571	0.345	0.943
10:00	0.303	6.029	0.272	1.098	0.668	0.429	0.173	1.363
12:00	0.050	11.048	0.272	1.098	0.819	0.229	0.600	0.524
14:00	0.239	6.762	0.388	0.863	0.331	0.971	0.463	0.734
16:00	0.162	7.905	0.433	0.784	1.000	0.000	0.345	0.943
18:00	0.796	2.371	0.583	0.549	0.819	0.229	0.463	0.734
20:00	0.293	6.143	0.347	0.941	0.407	0.829	0.028*	2.201
22:00	0.470	4.571	0.695	0.392	0.605	0.517	0.080	1.753
Area under the curve (AUC)	0.167	7.810	0.345	0.943	0.753	0.314		
STEPS per day	0.127	5.700						

Values reaching level of significance (p < 0.05; Bonferroni corrected for main effects of TIME p < 0.003 or p< 0.005 for lactate and heat rate) are indicated in red.

**Table 2:** Post hoc pairwise comparisons using Wilcoxon Test for heart rate, lactate, reaction time for ERT and DSST tasks of the Cognition test battery, as well as for respiratory exchange ratio (RER_peak_)

Heart rate @ 0 km/h					Heart rate @ 3 km/h					Heart rate @ 6 km/h					Heart rate@ 9 km/h				
Paired variables	N	T	Z	p	Paired variables	N	T	Z	p	Paired variables	N	T	Z	p	Paired variables	N	T	Z	p
BDC & ISO1	6	0	2.201	0.028	BDC & ISO1	6	0	2.201	0.028	BDC & ISO1	6	0	2.201	0.028	BDC & ISO1	6	4	1.363	0.173
BDC & ISO2/3	6	0	2.201	0.028	BDC & ISO2/3	6	0	2.201	0.028	BDC & ISO2/3	6	0	2.201	0.028	BDC & ISO2/3	6	1	1.992	0.046
BDC & ISO4	6	0	2.201	0.028	BDC & ISO4	6	0	2.201	0.028	BDC & ISO4	6	0	2.201	0.028	BDC & ISO4	6	0	2.201	0.028
BDC & R	6	8	0.524	0.600	BDC & R	6	9	0.314	0.753	BDC & R	6	7	0.734	0.463	BDC & R	6	5	1.153	0.249
ISO1 & ISO2/3	6	2	1.782	0.075	ISO1 & ISO2/3	6	2	1.782	0.075	ISO1 & ISO2/3	6	5	1.153	0.249	ISO1 & ISO2/3	6	1	1.992	0.046
ISO1 & ISO4	6	1	1.992	0.046	ISO1 & ISO4	6	0	2.201	0.028	ISO1 & ISO4	6	5	1.153	0.249	ISO1 & ISO4	6	0	2.201	0.028
ISO1 & R	6	1	1.992	0.046	ISO1 & R	6	2	1.782	0.075	ISO1 & R	6	4	1.363	0.173	ISO1 & R	6	10	0.105	0.917
ISO2/3 & ISO4	6	10	0.105	0.917	ISO2/3 & ISO4	6	1	1.992	0.046	ISO2/3 & ISO4	6	8	0.524	0.600	ISO2/3 & ISO4	6	8	0.524	0.600
ISO2/3 & R	6	0	2.201	0.028	ISO2/3 & R	6	1	1.992	0.046	ISO2/3 & R	6	1	1.992	0.046	ISO2/3 & R	6	0	2.201	0.028
ISO4 & R	6	0	2.201	0.028	ISO4 & R	6	0	2.201	0.028	ISO4 & R	6	0	2.201	0.028	ISO4 & R	6	1	1.992	0.046

Lactate @ 0 km/h					Lactate @ 3 km/h					RER_peak_									
Paired variables	N	T	Z	p	Paired variables	N	T	Z	p	Paired variables	N	T	Z	p					
BDC & ISO1	6	1.5	1.887	0.059	BDC & ISO1	6	9	0.314	0.753	BDC & ISO1	6	5	1.153	0.249					
BDC & ISO2/3	6	4	1.363	0.173	BDC & ISO2/3	6	9	0.314	0.753	BDC & ISO2/3	6	0	2.201	0.028					
BDC & ISO4	6	8	0.524	0.600	BDC & ISO4	6	7	0.734	0.463	BDC & ISO4	6	1	1.992	0.046					
BDC & R	6	6	0.943	0.345	BDC & R	6	1	1.992	0.046	BDC & R	6	0	2.201	0.028					
ISO1 & ISO2/3	6	8	0.524	0.600	ISO1 & ISO2/3	5	5	0.674	0.500	ISO1 & ISO2/3	6	0	2.201	0.028					
ISO1 & ISO4	6	7	0.734	0.463	ISO1 & ISO4	6	3	1.572	0.116	ISO1 & ISO4	5	0	2.023	0.043					
ISO1 & R	6	0	2.201	0.028	ISO1 & R	6	4	1.363	0.173	ISO1 & R	6	0	2.201	0.028					
ISO2/3 & ISO4	5	0	2.023	0.043	ISO2/3 & ISO4	6	10	0.105	0.917	ISO2/3 & ISO4	6	6.5	0.839	0.402					
ISO2/3 & R	6	5	1.153	0.249	ISO2/3 & R	6	8	0.524	0.600	ISO2/3 & R	6	6	0.943	0.345					
ISO4 & R	6	8	0.524	0.600	ISO4 & R	6	10	0.105	0.917	ISO4 & R	6	10	0.105	0.917					

ERT Reaction Time						DSST Reaction Time														
Paired variables	N	T	Z	p	Paired variables		N	T	Z	p										
BDC & ISO1	6	6	0.943	0.345	BDC & ISO1		6	6	0.943	0.345										
BDC & ISO2	6	0	2.201	0.028	BDC & ISO2		6	4	1.363	0.173										
BDC & ISO3	6	0	2.201	0.028	BDC & ISO3		6	0	2.201	0.028										
BDC & ISO4	6	0	2.201	0.028	BDC & ISO4		6	0	2.201	0.028										
BDC & R	5	0	2.023	0.043	BDC & R		5	0	2.023	0.043										
ISO1 & ISO2	6	5	1.153	0.249	ISO1 & ISO2		6	5	1.153	0.249										
ISO1 & ISO3	6	0	2.201	0.028	ISO1 & ISO3		6	0	2.201	0.028										
ISO1 & ISO4	6	0	2.201	0.028	ISO1 & ISO4		6	0	2.201	0.028										
ISO1 & R	5	0	2.023	0.043	ISO1 & R		5	0	2.023	0.043										
ISO2 & ISO3	6	0	2.201	0.028	ISO2 & ISO3		6	2	1.782	0.075										
ISO2 & ISO3	6	0	2.201	0.028	ISO2 & ISO3		6	0	2.201	0.028										
ISO2 & R	5	0	2.023	0.043	ISO2 & R		5	0	2.023	0.043										
ISO3 & ISO4	6	9	0.314	0.753	ISO3 & ISO4		6	0	2.201	0.028										
ISO3 & R	5	3	1.214	0.225	ISO3 & R		5	5	0.674	0.500										
ISO4 & R	5	3	1.214	0.225	ISO4 & R		5	5	0.674	0.500										

Values reaching level of significance (p < 0.05) are indicated in red

### Physical performance

**Fig. 3:**
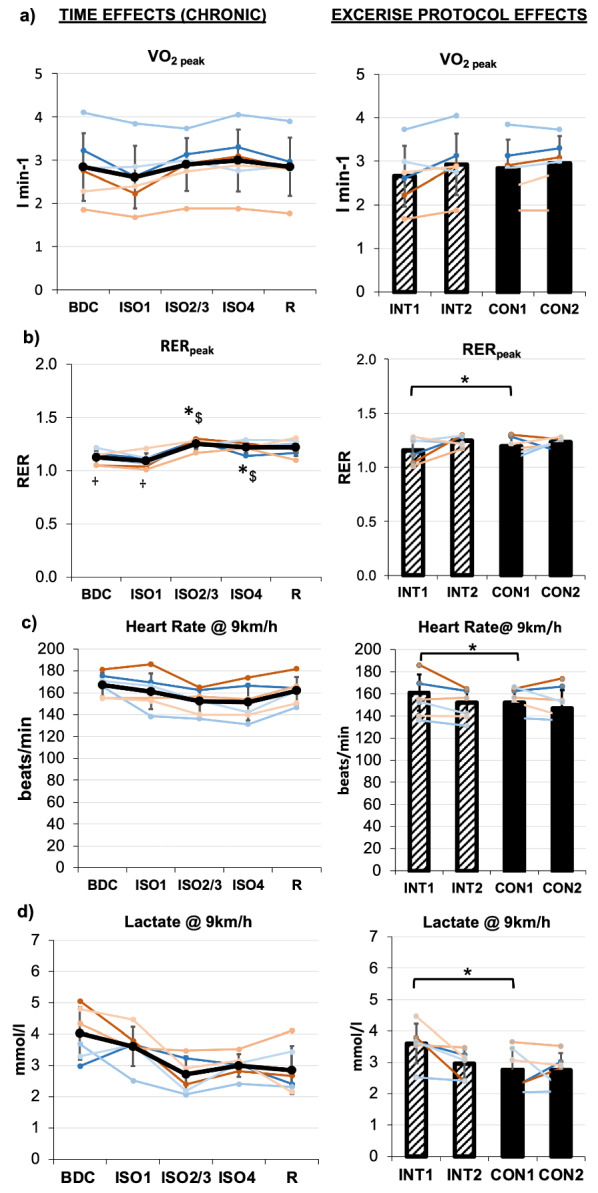
**a** Results of physical performance test regarding peak oxygen uptake (VO2_peak_), **b** peak respiratory exchange ratio (RER_peak_), **c** heart rate @ 9 km/h and 3d) lactate @ 9 km/h. Chronic effects over TIME are displayed on the left, exercise PROTOCOL effects on the right, respectively. Means are indicated in black line or bar graphs, female individuals are shown in red shades, male individuals in blue shades. TIME effects are presented for assessments during baseline data collection (BDC), at the beginning of isolation (ISO1), at midterm (ISO2/3), at the end (ISO4) and during the recovery phase (R). For exercise PROTOCOL effects comparing interval (INT, stripped bars) and continuous (CON, full bars) protocols, the mean at the beginning (INT1, CON1) and end (INT2, CON2) of each protocol is displayed. Error bars indicate standard deviation. Significant differences to BDC are indicated by *, to ISO1 by $, and to R by **†**

VO_2peak_ did not reveal an effect of *TIME, PROTOCOL* or *acuteEXERCISE*. A carry-over effect was found between INT1 and CON1 (see Table 1, Fig. 3a).

RER_peak_ did show a main effect of *TIME,* and was higher at ISO2/3, ISO4 and R compared to BDC and ISO1 (see Tables 1 , 2, Fig. 3b. RER_peak_ did not differ between exercise protocols, but a carry-over effect was found.

Lactate concentration during physical performance stage tests at 3 and 9 km/h running speed did steadily decrease in the course of isolation as a main effect of *TIME*, but missing the Bonferroni corrected level of significance (see Table 1, Fig. 3d). Carry-over effects between exercise protocol periods from INT1 to CON1 were found for 3 and 9 km/h running speeds. No difference between exercise *PROTOCOLs* was found for lactate concentrations.

Heart rate during physical performance tests at 0, 3, 6, and 9 km/h revealed a main effect of *TIME.* Heart rate significantly decreased during isolation and increased almost back to baseline during R (see Tables 1, 2, Fig. 3c). No carry-over effects for heart rate were detected, and no *PROTOCOL* specific difference was found.

### Growth factors

**Fig. 4:**
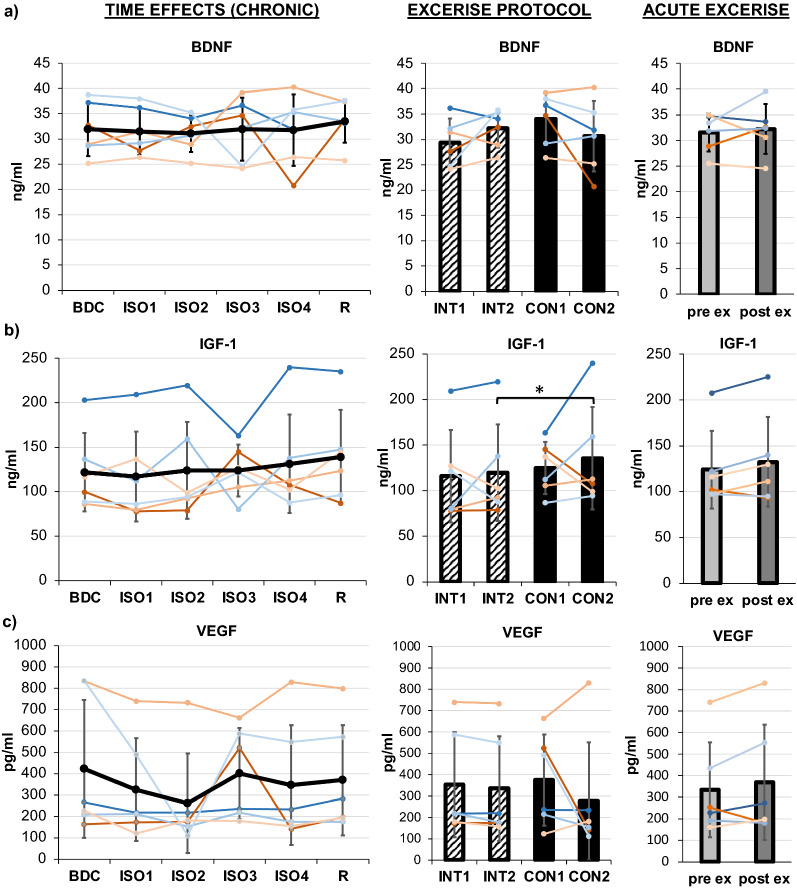
Results of growth factors BDNF (**a**), IGF-1 (**b**), and VEGF (**c**). Chronic effects over TIME are displayed on the left, exercise PROTOCOL effects in the middle, and acuteEXERCISE effect on the right. Means are indicated in black line or bar graphs, female individuals are shown in red shades, male individuals in blue shades. TIME effects are presented for assessments during baseline data collection (BDC), during isolation (ISO1-4), and during the recovery phase (R). For exercise PROTOCOL effects comparing interval (INT, stripped bars) and continuous (CON, full bars) protocols, the mean at the beginning (INT1, CON1) and end (INT2, CON2) of each protocol is displayed. AcuteEXERCISE effects from pre- (light grey) to post-exercise (dark grey) are shown. Error bars indicate standard deviation. Significant differences are indicated by *

BDNF did not show main effects of *TIME*, *PROTOCOL* or *acuteEXERCISE* (see Table 1, Fig. 4a).

IGF-1 concentration progressively increased over *TIME,* but marginally missed the Bonferroni corrected level of significance (p = 0.015; see Table 1 and Fig. 4b). There was no difference between the start of both training periods (no carry-over effect), but a significant and uniform difference between training *PROTOCOL*s with higher IGF-1 concentrations for CON compared to INT were detected. Comparisons of pre- and post-exercise measures indicated higher IGF-1 levels post-exercise but missing the level of significance (p = 0.052). VEGF did not show any effects for *TIME, PROTOCOL, and acuteEXERCISE* (see Table 1 and Fig. 4c). Post-exercise levels tended to be higher than pre-exercise levels (p = 0.076).

### Saliva cortisol

**Fig. 5:**
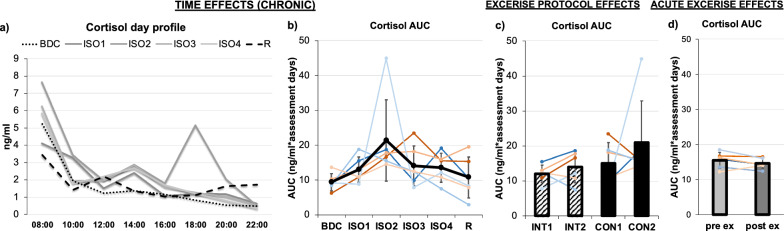
**a** Mean cortisol profile analyzed from saliva samples in two-hour intervals from 8:00 AM to 10:00 PM for the assessments over time during BDC (black dotted line), ISO1-4 (grey continuous lines), and R (black dashed line). **b** Mean area under the curve (AUC) of the cortisol profiles over time (BDC, ISO1-4, R). **c** For exercise PROTOCOL effects comparing interval (INT, stripped bars) and continuous (CON, full bars) protocols, the mean at the beginning (INT1, CON1) and end (INT2, CON2) of each protocol is displayed. **d** AcuteEXERCISE effects from pre- (light grey) to post-exercise (dark grey) are shown. Means in (**b**–**d**) are indicated in black line or bar graphs, female individuals are shown in red shades, male individuals in blue shades. Error bars indicate standard deviation

Day profile of saliva cortisol levels revealed elevated levels in the morning and afternoon during isolation compared to BDC and partly R, but no significant main effect of *TIME* was detected for the area under the curve as well as all time points of the day profile separately (see Table 1, Fig. 5a, b). No difference between exercise *PROTOCOLs* (Fig. 5c) or between pre- and post-exercise (*acuteEXERCISE*) samples was found for cortisol (Fig. 5d).

### Mood

**Fig. 6:**
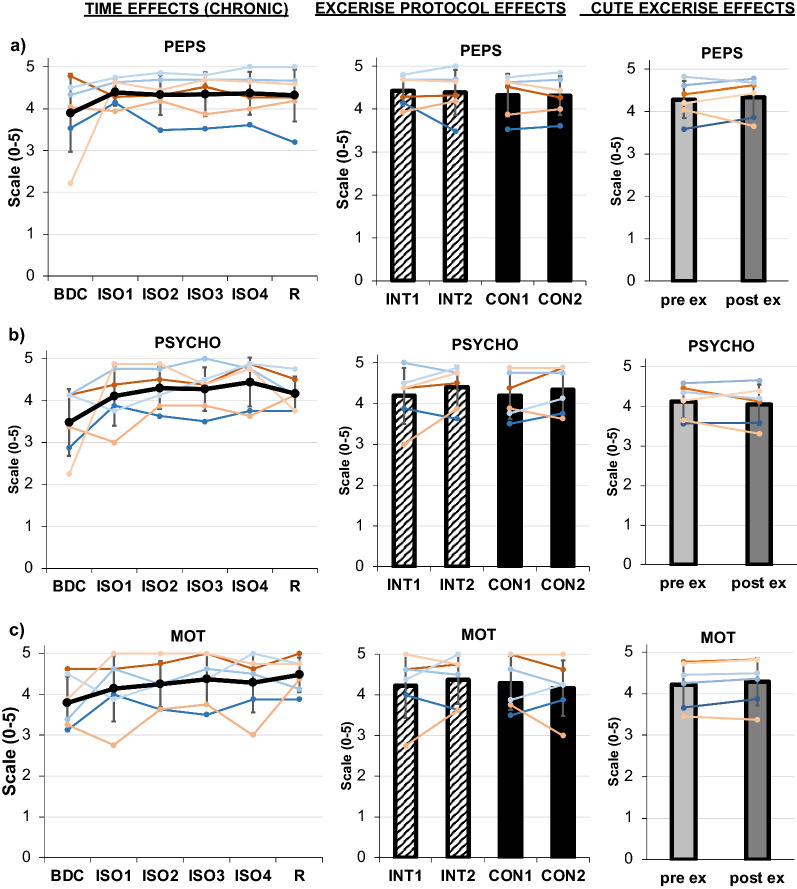
**a** Results of MoodMeter^®^: Physical state (PEPS), **b** psychological strain (PSYCHO), and **c** motivational state (MOT). Chronic effects over TIME are displayed in the left column, exercise PROTOCOL effects in the middle column, and acuteEXERCISE effect in the right column. Means are indicated in black line or bar graphs, female individuals are shown in red shades, male individuals in blue shades. TIME effects are presented for assessments during baseline data collection (BDC), during isolation (ISO1-4), and during the recovery phase (R). For exercise PROTOCOL effects comparing interval (INT, stripped bars) and continuous (CON, full bars) protocols, the mean at the beginning (INT1, CON1) and end (INT2, CON2) of each protocol is displayed. AcuteEXERCISE effects from pre- (light grey) to post-exercise (dark grey) are shown. Error bars indicate standard deviation

The MoodMeter^®^ questionnaire revealed no significant effects of *TIME, PROTOCOL or acuteEXERCISE* for any of the three dimensions PEPS, PSYCHO, and MOT, but the mood was generally observed to increase from BDC to in isolation (see Table 1 and Fig. 6a–c).

### Affect

**Fig. 7:**
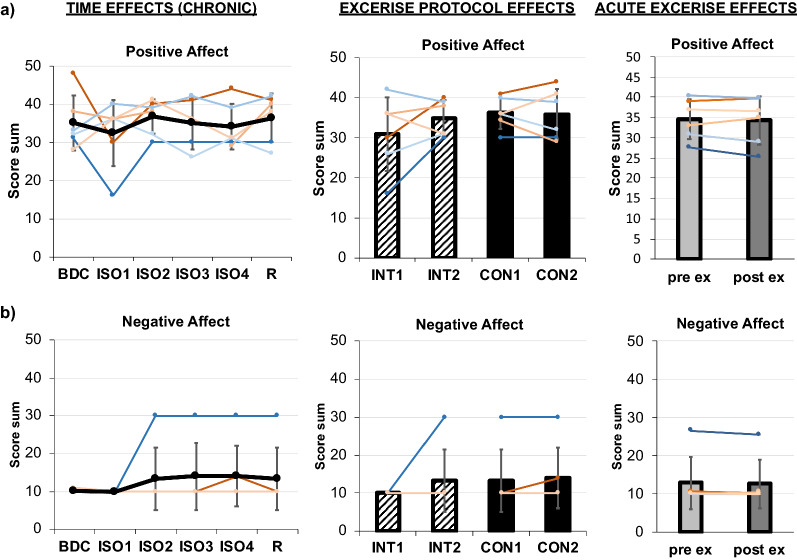
Results of the PANAS questionnaire: Positive Affect (**a**), Negative Affect (**b**). Chronic effects over TIME are displayed on the left, exercise PROTOCOL effects in the middle, and acuteEXERCISE effect on the right. Means are indicated in black line or bar graphs, female individuals are shown in red shades, male individuals in blue shades. TIME effects are presented for assessments during baseline data collection (BDC), during isolation (ISO1-4), and during recovery phase (R). For exercise PROTOCOL effects comparing interval (INT, stripped bars) and continuous (CON, full bars) protocols, the mean at the beginning (INT1, CON1) and end (INT2, CON2) of each protocol is displayed. AcuteEXERCISE effects from pre- (light grey) to post-exercise (dark grey) are shown. Error bars indicate standard deviation

No effect of *TIME*, *PROTOCOL* or *acuteEXERCISE* for General Positive Affect or Negative Affect Sum was found (see Table 1 and Fig. 7a, b). It has to be noted that all but one participant mostly reported to perceive negative affect "not at all", almost without variation.

### Cognition

**Fig. 8:**
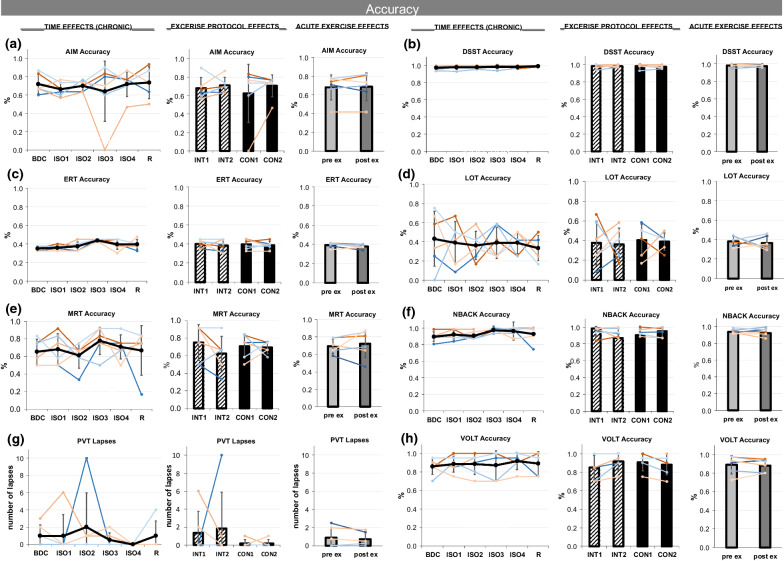

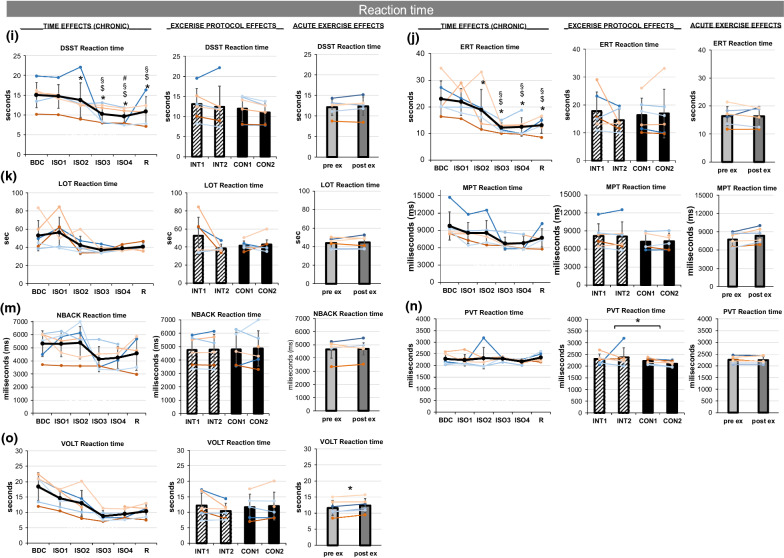
**a**–**h** Accuracy within the Cognition tests in alphabetical order: **a** AIM accuracy, **b** DSST accuracy, **c** ERT accuracy, **d** LOT accuracy, **e** MRT accuracy, **f** NBACK accuracy, **g** PVT lapses, **h** VOLT accuracy. Presented in each figure in columns are chronic effects over TIME, exercise PROTOCOL effects, and acute EXERCISE effects. TIME effects are presented for assessments during baseline data collection (BDC), during isolation (ISO1-4), and during the recovery phase (R). For exercise PROTOCOL effects comparing interval (INT, stripped bars) and continuous (CON, full bars) protocols, the mean at the beginning (INT1, CON1) and end (INT2, CON2) of each protocol is displayed. Acute EXERCISE effects from pre- (light grey) to post-exercise (dark grey) are shown. Means are indicated in black line or bar graphs, female individuals are shown in red shades, male individuals in blue shades. Error bars indicate standard deviation.  8**i**–**o** Reaction times within the Cognition tests in alphabetical order: **i** DSST reaction time, **j** ERT reaction time, **k** LOT reaction time, **l** MPT reaction time, **m** NBACK reaction time, **n** PVT reaction time, **o** VOLT reaction time. Presented in each figure in columns are chronic effects over TIME, exercise PROTOCOL effects, and acute EXERCISE effects. TIME effects are presented for assessments during baseline data collection (BDC), during isolation (ISO1-4), and during the recovery phase (R). For exercise PROTOCOL effects comparing interval (INT, stripped bars) and continuous (CON, full bars) protocols, the mean at the beginning (INT1, CON1) and end (INT2, CON2) of each protocol is displayed. Acute EXERCISE effects from pre- (light grey) to post-exercise (dark grey) are shown. Means are indicated in black line or bar graphs, female individuals are shown in red shades, male individuals in blue shades. Error bars indicate standard deviation. For TIME effects, significant differences to BDC are indicated by *, to ISO1 by $, to ISO2 by §, and to ISO3 by #. For exercise PROTOCOL and acuteEXERCISE effects, significant differences are marked by *.

The accuracy of performing the Cognition test battery remained stable for AIM, DSST, LOT, MRT, NBACK and VOLT (Fig. 8a, b, d–h), but showed slight improvements ERT (p = 0.055, Fig. 8c). The reaction time of the tests DSST, ERT, LOT, MPT, NBACK, and VOLT (Fig. 8i–m & o) progressively decreased over *TIME* (significantly for DSST and ERT: p = 0.001; see Table 1, Fig. 8i, j)). PVT reaction time (Fig. 8n) and lapses (Fig. 8 g) did not change over time. Significant lower reaction time for PVT was found for CON compared to INT (Fig. 8n). Lower reaction time was found for MPT (Fig. 8l) and VOLT (Fig. 8o) pre-exercise compared to post-exercise. No further pre- to post-exercise differences and no carry-over effects (INT1 vs. CON1) were found.

## Discussion

This study aimed to explore the effect of exercise on physical and cognitive performance, mood and affect as well as hormonal changes during a long-duration ICE mission. Intensity-matched INT and CON running exercise protocols and acute exercise effects have been examined and individually presented to promote optimization and individualization of exercise interventions for living under ICE or pandemic conditions.

The results suggest that subjective mood and affect state as well as cognitive performance, BDNF and VEGF level were unchanged during a four-month space-analogue isolation mission with accompanying regular and efficient running exercise. Physical performance, and reaction time during cognitive testing improved throughout the intervention, while basal IGF-1 level tended to increase. Clear acute exercise effects (tested pre and post exercise on two separate days and times) could not be observed, but reaction time in regard of sensorimotor speed and memory for complex figures was found to be slower after exercise. Higher IGF-1 concentrations and lower reaction time during the psychomotor vigilance test were found for the intensity-matched CON compared to the INT running protocol. However, carry-over effects found for the physical fitness parameter indicate, that the wash-out period between exercise protocols was insufficient.

### Physical fitness

RER_peak_, heart rate and lactate values (VO_2peak_ tended) during physical performance tests improved in the course of isolation. Accordingly, the physical fitness of the crewmembers improved as a result of the individual regular running protocol. However, at ISO4 the improvement of physical performance parameters was flattened or stopped, which indicates that the exercise protocols were not effective at this time anymore. This could be due to completed adaptation and insufficient progression of training volume. In respect of the actigraphy data and in accordance with Koschate et al. [17], it could further be assumed that decreasing overall physical activity during isolation contributes to this stagnation of physical fitness. During R, heart rate and VO_2peak_ tended back to baseline level whereas lactate and RER_peak_ more or less remained on improved levels. The results highlight the need for maintained daily physical activity levels and/or progressive regular as well as individualized exercise protocols during extended missions (> three months) in order to counteract declining physical activity levels and physical deteriorations.

The present study was not able to deliver clear results in favor of INT or CON exercise protocol in regard to the present physical performance parameters as confirmed by Koschate et al. [17], which is suggested to be due to the short duration of each training protocol (24 training days) and a short “wash-out” period (fifteen days) between protocols referring to the carry-over effects for lactate, VO_2peak_, and RER_peak_. This, together with the intensity-matched approach, is suggested to mainly contribute to missing exercise protocol effects for the following investigated parameters.

### Mood, affect, and cognition

Perceived mood and affect were stable in the present study over time with the regular exercise regimen and therewith extends previous short duration studies (30 to 45 days, 10, 11, 44). There is a growing body of evidence for the beneficial effect of exercise on mental health in general [7, 8], but also especially in relation to isolation and confinement, such as during the COVID-19 pandemic [51–53] as well as to space and space-analogue conditions [1, 9–11, 44, 54]. Habitually exercising participants were able to maintain their state of mood during isolation and confinement studies of up to nine months, whereas sedentary individuals showed substantial impairments [9–11]. Lower levels and restrictions of physical activity and exercise were found to correlate with impaired sleep, mood, and affect [10, 11, 53]. The study by Abeln et al. [9] also points out that participants voluntarily increased the exercise volume during the most difficult mid-winter time during Antarctic overwintering, which might be a sign of the higher need and higher perceived benefit of exercise to cope with this extreme situation. Higher physical activity level is known to positively correlate with positive affect and negatively with negative affect and stress [55]. Here, it can only be speculated based on these previous studies, that the maintained state of mood is correlated to the efficient regular exercise intervention and that mood would have shown a more negative progression if physical activity or fitness level declined. It supports the notion that regular exercise is an important tool to chronically prevent mood impairments, not only in ICE environments. However, individual coping strategies, feelings of loneliness, lack of social support, and crew cohesion have also been discussed as potential factors amongst others for previously found heterogenous mood and affect state progressions [1], which are out of the scope of this manuscript.

Considering the benefit of physical activity on cognition as well as the link between positive emotions with cognitive performance [56], the reaction time of the cognitive tasks was improving, and accuracy was stable as expected. Baser et al. [49] found similar progressions for the different tests and also higher variance for the LOT and MRT. The present results replicate their findings of practice and test-retests effects, which have to be taken into account and might confound isolation or exercise dependent effects. According to the comparable outcomes, the investigated domains of cognitive functions with high importance for astronauts can be assumed to be unimpaired during four months of isolation with regular exercise training and stable states of mood and affect. On an individual level, there are two individuals, one male and one female, who generally rate their state of mood (PEPS, PSYCHO and MOT) less positive and more likely vary in their cognitive performance. This observation might display the link between mood and cognition (e. g. lower motivation leads to lower cognitive performance), but there was no negative progression visible for those participants with accompanying exercise intervention. While this supports the notion of chronic exercise benefits, acute exercise effects comparing pre- and post-exercise timepoints should not be neglected. (Only) Two tasks (VOLT and MPT) revealed slower reaction time immediately after exercise, while accuracy was not impaired. Therefore, it requires further investigation whether these findings can be confirmed, are meaningful and if other exercise protocols might be more beneficial.

Contrary to previous studies finding acute benefits of exercise on cognition, mood and affect, it can be assumed that, here, separate assessment days for pre- and post-exercise comparisons might have contributed to this missing acute exercise effect. Different factors, such as daytime (circadian) effects as well as different psycho-physiological states due to training, work and nutrition schedule as well as social interactions or sleep cannot be ruled out to influence the pre- to post-exercise comparison. Separate days instead of testing immediately prior to and after exercise might mitigate repetition/practice and pre-exercise level effects for cognitive testing [57], as well as potential memory, expectation or placebo effects on mood and affect [58–60], which is a strength of the present study.

No difference between INT and CON exercise protocols could be observed in regard of mood, and affect, and for cognitive performance (besides for the PVT) which might be explained by their intensity-matched design, whereas previous studies mostly found differences when comparing moderate continuous and high-intensity interval protocols [15, 61–64]. But again, the short protocol duration and insufficient wash-out phase of the present study should also be taken into account. Nonetheless, the faster reaction time during the psychomotor vigilance test for CON compared to INT is an important outcome in terms of exercise recommendations not only for ICE missions. There is one individual who mainly accounts for the slower reaction time at INT2, but the effect is still present, when this individual is excluded. With all due respect, this exercise PROTOCOL effect for the reaction time in the PVT should be interpreted with caution.

### Cortisol

Elevated cortisol levels have repeatedly been observed in previous space, and confinement observation studies [10, 36, 37, 44] and were suggested to reflect the underlying mechanism for the successful adaptation to isolation conditions as no accompanying cognitive or psychological impairments could be observed. Not only isolation but also exercise should be taken into account for cortisol levels. Particularly high-intensity (> 60% of VO2_peak_ [65]) and/or high-volume exercise was shown to higher cortisol levels [66–70] and higher cortisol levels were also observed for higher compared to lower volume of exercise protocols in isolation [10, 11]. Previously, elevated cortisol levels were found at the start of isolation, but also at the time when exercise protocol commenced.

Here, no significant chronic or acute effect of isolation and/or exercise on cortisol level was observed. On a descriptive level, cortisol level tends to be higher during isolation, in particular in the morning and afternoon, compared to non-isolated baseline and recovery conditions but were found to be still within the normal range (instructions salivary cortisol ELISA by DRG Instruments: morning < 19.8 ng/mL; noon < 12.7 ng/mL; evening < 4.0 ng/mL). There are many differences between the previous isolation study in the Human Exploration Research Analog (HERA) in Houston, Texas, to the present isolation study in NEK in Moscow, such as size of the facility, operational tasks, crew size and composition, privacy space, and duration of isolation amongst others and besides the exercise protocol.

Accordingly, the present exercise intervention during isolation seems to be appropriate to maintain cortisol levels. Exercise-induced cortisol responses should be further investigated and distinguished from other stress responses during isolation and confinement studies.

### Neurotrophic and growth factors

Contrary to the findings by Seifert et al. [71], BDNF and VEGF were not found to be (chronically) altered during isolation or with aerobic training and fitness. Previous literature about the effect of exercise on BDNF, IGF-1, and VEGF expression is heterogeneous and discussed to be dependent on exercise protocols, e. g. exercise type, intensity, volume [24, 29, 34, 67, 72–74] as well as the level of physical fitness [24, 75]. Consequently, moderate exercise intensity and increasing level of fitness in the present study might have contributed to the missing increase in basal BDNF and VEGF concentration.

IGF-1 is known to coordinate brain responses to metabolic status, muscle mass, and body homeostasis and to trigger cell proliferation and protect cell apoptosis [76]. The majority of existing studies have demonstrated its release triggered by aerobic exercise [29–34]. This supports the notion that the slightly rising basal levels of IGF-1 here are triggered by aerobic exercise. Increased levels of IGF-1 might coordinate physical adaptation (in reference to physical performance variables). Furthermore, IGF-1 can cross the blood–brain barrier and has been correlated with hippocampus volume and cognitive performance [77], which is why it can be speculated that increased basal IGF-1 levels might also be responsible for the maintenance/protection of cognitive performance [28, 78]. This is supported by similar findings regarding IGF-1 level observed in previous short-duration space-analogue isolation missions with regular exercise training along with preserved cognitive performance, mood and state of affect [10, 11, 44]. Interestingly, cell culture and animal studies provide evidence for an additive effect and higher neurotrophic impact of BDNF with higher IGF-1 presence [79]. According to this, there might be a chronic protective or a generative effect of exercise via the regulation and interplay of neurotrophic growth factors that this study design has not captured.

Something that should not be disregarded is the dip of IGF-1 in males at ISO3 was found in the two crew members who executed the extravehicular activities on the Lunar surface simulator and have been isolated from the rest of the crew for a period of time prior to ISO3 in a separate compartment. It can only be assumed that the reduced amount of physical activity and exercise during this period has contributed to this reduction in IGF-1, but this requires further investigation. The IGF-1 levels were back to normal at ISO4.

No *acuteEXERCISE* effect on neurotrophic and vascular-endothelial growth factors was detected comparing pre- with post-exercise data, but results marginally missed significance for IGF-1 (p = 0.052) and VEGF (p = 0.074). There is evidence for temporary, transient and local (central) exercise-induced releases of BDNF [24, 32, 80, 81], IGF-1 [82, 83] and VEGF [84, 85]. Moreover, day time/circadian variations were found for serum BDNF [86, 87] and for free serum IGF-1 levels [88], whereas no circadian variation was shown for VEGF [89, 90]. Blood draws for pre-exercise assessments took place in the morning, whereas post-exercise blood draw was executed about five minutes after exercise on a separate day at various daytimes. It should be noted that daytime dependent decreases might have diminished exercise-triggered increase, at least for BDNF and IGF-1. There is more reason to believe that exercise facilitates the effectiveness and turnover of these factors and that peripheral levels must not necessarily reflect central ones (brain as main source releasing BDNF [24, 80, 81, 85, 91]). This might explain missing acute exercise effects on these factors contrary to other studies [24, 33, 92]. Considering the circadian rhythm, facilitation, and additive effect of IGF-1, marginally higher IGF-1 concentrations post-exercise found here might be more significant as they visually and statistically reveal.

Although exercise *PROTOCOL* periods were reduced to four weeks respectively, interval compared to continuous exercise protocol was found to be significantly different in the effect on basal IGF-1 level, in which significant higher IGF-1 values were found at the end of the continuous training period (CON2) in all participants compared to the end of the interval training (INT2) without carry-over effect. This effect of the moderate-intensity continuous running protocol might be of added value for to date heterogenous findings regarding exercise recommendations for mental health [28]. However, in regard of the heterogeneity of changes from CON1 to CON2 as well as INT1 to INT2, confirmation is required in future studies. On the contrary to the findings by Saucedo Marquez et al. [81] and Santos et al. [73], no difference in BDNF level between exercise protocols could be observed here. High-intensity interval exercise in the previous studies might have contributed to this discrepancy [34, 73, 81]. Here, exercise intensity was aimed to be matched for both protocols for average treadmill speed. The present study delivers important results for intensity-matched comparison of interval and continuous exercise protocols.

The present findings suggest that regular individual aerobic running exercise promotes participants' fitness, cognitive performance, and perceived state of mood and affect during 120 days of isolation. This data, therefore, delivers more evidence for the benefit of individual aerobic exercise for physical and mental health and cognitive performance while living under ICE conditions for up to four months. While a chronic holistic benefit of exercise is assumed, the acute effect of exercise could not be supported, which is suggested to be mostly caused by separate pre- and post-exercise assessment days. This is an added-value of the present study, as repetition effects on two separate days are less likely to interfere. Moderate intensity continuous running seems to excel intensity-matched interval running regarding the release of IGF-1 and vigilance. Further studies are required to confirm the results for prolonged isolation and wash-out periods. Furthermore, future investigations should test other exercise protocols (various types, modalities, intensities, volumes) for augmented chronic and acute exercise effects and their hormonal response towards an effective and holistic countermeasure against psycho-physiological impairments during isolation.

### Limitations

Space and space analogue missions are of high effort and cost. Long-term investigations like the present 120 days isolation mission aim to mimic real space missions, including space limitations, people, load, contact to the outside, etc. Furthermore, the number of implemented studies is high in order to maximize the output, which requires consensus of involved parties and increases the potential of influencing factors. The advantage is the high level of control because almost everything is scheduled, predetermined and monitored. Consequently, the small sample size of a total of six participants and three individuals per sex has to be taken into account. A non-isolated or non-exercising control group is missing. The authors would like to emphasize at this point, that, because of the missing control group, the results cannot clearly be attributed to exercise. This interpretation is based on previous studies [9–11, 53].

Furthermore, it was difficult to schedule the present assessments in the dense implemented protocol as required. Exercise and subsequent test sessions were individually scheduled at various times during the day allowing only one participant at a time. The four-week training period per protocol might have been too short of revealing significant *PROTOCOL* effects. Moreover, the "wash-out" period (fifteen days in-between protocols) was too short as many carry-over effects (comparing CON1 and INT1) were found, which might have blurred protocol specific effects in this cross-over design. Blood for pre-exercise and chronic effect assessment was always drawn in the morning after wake-up (see chapter 2.2.) in order to obtain basal levels and avoid influences of nutrition or circadian cycle. The disadvantage is the missing data immediately prior to exercise. Due to ethical and economic reasons and implementation negotiations with mission control and other principal investigators, additional blood draws were not possible. Regarding cognition and mood, two assessments on the same day for direct pre- versus post-exercise comparisons regarding cognition and mood were avoided to control for effects of repetition, bias and social expectancy. While all this might have contributed to divergent results compared to previous space and exercise studies and should be taken into account, the present observational study does deliver important, comprehensive and (approximately) holistic data about the chronic and acute effects of isolation with regular exercise training of two different modalities over a 120 day period.

## Conclusion

The present findings suggest that 120 days of isolation and confinement can be undergone without cognitive and mental deteriorations. The present efficient individual aerobic running training is assumed to play an important role in this regard chronically, whereas no acute benefits of exercise could be detected. Continuous running exercise was found to trigger higher IGF-1 expression and vigilance than intensity-matched interval running, although both protocols were only executed for four weeks, respectively, and separated by an insufficient wash-out period. Systematic and prolonged investigations are required to follow up on exercise-protocol specific differences in order to optimize the exercise intervention for long-term psycho-physiological health and well-being.

## Supplementary Information

**Additional file 1:** Example for continuous training (CON, upper figure) and interval training (INT, lower figure) micro cycle. Day 1 of the training micro cycle is presented in blue, Day 2 in green, and day 3 in red color. The lag is provided on the x-axis in seconds (s) and treadmill on the y-axis in kilometer per hours (km h^-1^).

## Data Availability

Data generated or analyzed during this study are included in this published article [and its supplementary information files]. Datasets used during the current study are available from the corresponding author on reasonable request.

## Abbreviations

AcuteEXERCISE: Acute exercise effects
AIM: Abstract imaging matching task: BDC: Baseline data collection
BDNF: Brain-derived neurotrophic factor
CON: Continuous exercise
DSST: Digit-symbol substitution task
ERT: Emotion recognition task
ICE: Isolated, confined, extreme
IGF-1: Insulin-like growth factor 1
IBMP: Institute of Biomedical Problems
INT: Interval exercise
ISO: Isolation
LOT: Line orientation task
MD: Mission day
MOT: Perceived motivational state
MRT: Matrix reasoning task
MPT: Motor praxis task
NASA HRP: National Aeronautics and Space Administration Human Research Program
NBACK: N-Back task
NEK: Nezemnyy Eksperimental'nyy Kompleks
R: Recovery phase
SIRIUS-19: Scientific International Research in Unique Terrestrial Station 2019
PANAS: Positive affect and negative affect schedule
PEPS: Perceived physical state
PROTOCOL: Exercise protocol effects
PSYCHO: Perceived psychological strain
PVT: Psychomotor vigilance test
RAS: Russian Academy of Sciences
RER_peak_: Peak respiratory exchange ratio
TIME: Chronic effects over time
VEGF: Vascular-endothelial growth factor
VOLT: Visual Object Learning Task
VO_2peak_: Peak oxygen uptake

## Footnotes

1 The actigraphy data was shared with another group, who recorded actigraphy for other purposes, which is why not the same amount of data was stored for all participants. However, this data set delivers important data to estimate the physical activity behavior of the crewmembers in the course of isolation.
