# Supplementary material for: Chronic, acute and protocol-dependent effects of exercise on psycho-physiological health during long-term isolation and confinement
Source: BMC Neurosci. 2022 Jun 30;23:41. doi: 10.1186/s12868-022-00723-x (PMC9244384; doi:10.1186/s12868-022-00723-x)
Supplement: Supplementary file 1 — Additional file 1: Example for continuous training (CON, upper figure) and interval training (INT, lower figure) micro cycle. Day 1 of the training micro cycle is presented in blue, Day 2 in green, and day 3 in red color. The lag is provided on the x-axis in seconds (s) and treadmill on the y-axis in kilometer per hours (km h-1). [file 12868_2022_723_MOESM1_ESM.docx]

**Supplementary files**

**
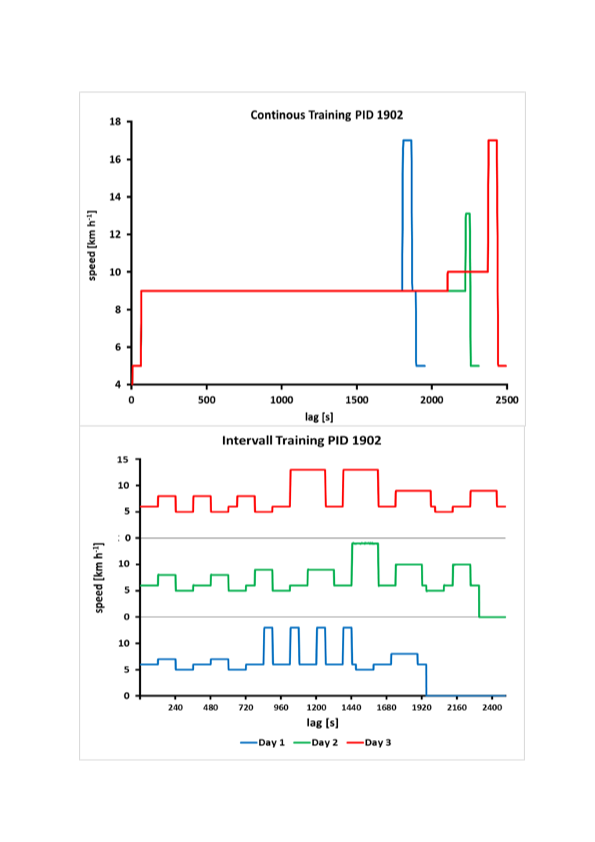
**

Figure 9: Example for continuous training (CON, upper figure) and interval training (INT, lower figure) micro cycle. Day 1 of the training micro cycle is presented in blue, Day 2 in green, and day 3 in red color. The lag is provided on the x-axis in seconds (s) and treadmill on the y-axis in kilometer per hours (km h^-1^).
